# Firing activity of locus coeruleus noradrenergic neurons decreases in necdin-deficient mice, an animal model of Prader–Willi syndrome

**DOI:** 10.1186/s11689-020-09323-4

**Published:** 2020-07-29

**Authors:** Rui-Ni Wu, Wei-Chen Hung, Ching-Tsuey Chen, Li-Ping Tsai, Wen-Sung Lai, Ming-Yuan Min, Shi-Bing Wong

**Affiliations:** 1 Department of Pediatrics, Taipei Tzu Chi Hospital, Buddhist Tzu Chi Medical Foundation, No. 289, Jiangguo Rd, Xindian Dist, New Taipei City, 23142 Taiwan; 2 Department of Life Science, College of Life Science, National Taiwan University, No. 1, Sec 4, Roosevelt Rd, Taipei, 10617 Taiwan; 3 School of Medicine, Tzu Chi University, No. 701, Sec 3, Jhongyang Rd, Hualien, 97071 Taiwan; 4 Department of Psychology, National Taiwan University, No. 1, Sec 4, Roosevelt Rd, Taipei, 10617 Taiwan

**Keywords:** Necdin, Prader–Willi syndrome, Hypotonia, Hypercapnia, Locus coeruleus, A-type potassium current

## Abstract

**Background:**

Prader–Willi syndrome (PWS) is a neurodevelopmental disorder characterized by multiple respiratory, cognitive, endocrine, and behavioral symptoms, such as central apnea, intellectual disabilities, exaggerated stress responses, and temper tantrums. The locus coeruleus noradrenergic system (LC-NE) modulates a diverse range of behaviors, including arousal, learning, pain modulation, and stress-induced negative affective states, which are possibly correlated with the pathogenesis of PWS phenotypes. Therefore, we evaluated the LC-NE neuronal activity of necdin-deficient mice, an animal model of PWS.

**Methods:**

Heterozygous necdin-deficient mice (B6.Cg-Ndn^tm1ky^) were bred from wild-type (WT) females to generate WT (+m/+p) and heterozygotes (+m/−p) animals, which were examined of LC-NE neuronal activity, developmental reflexes, and plethysmography.

**Results:**

On slice electrophysiology, LC-NE neurons of *Ndn*^*tm1ky*^ mice with necdin deficiency showed significantly decreased spontaneous activities and impaired excitability, which was mediated by enhanced A-type voltage-dependent potassium currents. *Ndn*^*tm1ky*^ mice also exhibited the neonatal phenotypes of PWS, such as hypotonia and blunt respiratory responses to hypercapnia.

**Conclusions:**

LC-NE neuronal firing activity decreased in necdin-deficient mice, suggesting that LC, the primary source of norepinephrine in the central nervous system, is possibly involved in PWS pathogenesis.

## Background

Prader–Willi syndrome (PWS) is a neurodevelopmental disorder characterized by multiple endocrine, metabolic, respiratory, cognitive, and behavioral/psychiatric symptoms. Affected infants uniformly have significant hypotonia, feeding difficulties, central apneas, and failure to thrive, followed by excessive appetite in later infancy or early childhood with gradual development of obesity, short stature, intellectual disabilities, and behavioral problems [1, 2]. Although PWS is well-known as a genetic obesity syndrome, care for patients with PWS poses significant challenges and burden to caregivers and to the society predominantly because of their neurodevelopmental disabilities [3–6]. The exaggerated stress responses and temper tantrums were the symptoms that most affected the quality of life of patients with PWS in adulthood [4, 7].

PWS is caused by the loss of expression of imprinted, paternally inherited genes on chromosome 15q11.2-q13, including five functional genes (*MKRN3*, *MAGEL2*, *NDN*, *NPAP1*, and *SNURF-SNRPN*) and a cluster of small nucleolar RNA genes. The lost expression of these functional genes results in a wide spectrum of clinical phenotypes of PWS [8]. Necdin, encoded by *NDN*, plays an integral role in mitotic arrest, differentiation, and survival of postmitotic neurons and is highly expressed in the forebrain, raphe nucleus, and locus coeruleus (LC) [9]. Therefore, necdin deficiency results in widespread abnormalities of the parts of the nervous system, such as brainstem noradrenergic neurons [10], forebrain GABAergic neurons [9], and medullary serotonergic neurons [11, 12]. Phenotypically, necdin-deficient mice could exhibit several key symptoms of PWS, including sensory motor defects, altered pain threshold, and congenital hypoventilation [13]. As brainstem serotonergic neurons play important roles in respiratory maturation and regulation, studies using *Ndn*^*tm1-Mus*^ mice showed that necdin deficiency led to serotonergic neuroarchitectural changes and increased spontaneous firing of serotonergic neurons, which led to increased expression and activity of serotonin transporters [12]. Altered medullary serotonergic system structure and function were also possible causes of sudden infant mortality syndrome, which endangered children with PWS [14, 15]. These findings support the hypothesis that brainstem serotonopathy is part of the pathophysiology of the respiratory and behavioral symptoms of PWS; however, other neuromodulatory neurons (e.g., noradrenergic and dopaminergic) closely interact with each other to maintain vital cognitive and behavioral function, which may also influence the pathogenesis of PWS [16, 17].

The locus coeruleus (LC) is a small, tightly packed pontine brain region with numerous projections to the forebrain and spinal cord. The LC neurons are the primary resource of norepinephrine (NE) in the CNS; along with serotonergic, dopaminergic, and cholinergic systems, these neuromodulatory systems support cognitive function in higher organisms such as attention, emotion, goal-directed behavior, and decision-making derive [16]. Moreover, the LC neurons play an important role in maintaining stable respiration in young animals by enhancing carbon dioxide (CO_2_) sensitivity [18]. The output from LC neurons also maintains adequate muscle tone [19, 20]. Notably, this spectrum of physiological functions closely overlaps with several key manifestations of PWS, such as abnormality of arousal, altered pain perception, intellectual disabilities, exaggerated stress responses, hypotonia, and blunted ventilatory responses to hypercapnia [7, 21–23]. Therefore, in this study, we investigated the LC-NE neuronal activities of *Ndn*^*tm1ky*^ mice as well as the developmental reflexes and ventilatory responses to hypercapnia associated with neonatal symptoms of PWS. Our study provided evidence that *Ndn*^*tm1ky*^ mice recapitulated PWS phenotypes, and LC-NE neuronal dysfunction may be part of pathophysiology of PWS.

## Methods

### Animals

Heterozygous necdin-deficient mice (B6.Cg-Ndn^tm1ky^) were purchased from RIKEN (Saitama, Japan). *Ndn* was maternally imprinted, and only the paternal allele was functional. Therefore, heterozygous males carrying an NDN-deleted allele were bred from wild-type (WT) females (ICR background) to generate WT (+m/+p) and heterozygotes (+m/−p) animals in which the paternal allele was deleted, leading to necdin deficiency. These animals were used in all experiments. The use of animals was approved by the Ethical Committee for Animal Research of the Buddhist Taipei Tzu Chi General Hospital (105-IACUC-017) and was in accordance with the National Institutes of Health guidelines. Every effort was made to minimize the number of animals used and their suffering.

### Immunohistochemistry

Mice were anesthetized with urethane (1.3 g/kg) via intraperitoneal injection and perfused transcardially through the left ventricle with saline, followed by 0.1 M phosphate buffer (PB, pH 7.4) containing 4% paraformaldehyde. The brain was dissected and fixed overnight in the same fixation solution at 4 °C and stored in 30% sucrose in 0.05 M PB. To evaluate the necdin expression in the CNS, serial coronal sections were cut into 50-μm-thick sections using a freezing microtome. Slices were incubated for 1 h at room temperature in phosphate-buffered saline containing 0.03% Triton X-100 (PBST), 2% bovine serum albumin, and 10% normal goat serum and then incubated overnight at 4 °C in PBST containing 1/1000 dilution of rabbit antibodies against necdin (Bio Academia, Osaka, Japan) for 40 h at 4 °C. After rinsing with PBST, tissue slices were incubated with the secondary antibodies for 3 h: 1/200 dilution of goat anti-rabbit IgG-conjugated Alexa Fluor 594 (Jackson ImmunoResearch, West Grove, PA, USA). After rinsing with PB, the slices were mounted with RapiClear 1.47 (SunJin Lab, Hsinchu City, Taiwan), and a cover slip was placed. Immunofluorescence images were observed using a Leica SP8 confocal microscope (Leica microsystems, Wetzlar, Germany).

### Preparation of brainstem slices

The animals were anesthetized with 5% isoflurane in O_2_ and decapitated. The brains were rapidly exposed and chilled with ice-cold artificial cerebrospinal fluid (ACSF) with 119 mM NaCl, 2.5 mM KCl, 1.3 mM MgSO_4_, 26.2 mM NaHCO_3_, 1 mM NAH_2_PO_4_, 2.5 mM CaCl_2_, and 11 mM glucose, oxygenated with 95% O_2_ and 5% CO_2_ at pH 7.4. Coronal brainstem slices (300 μm) harboring LC were prepared using a vibratome (VT1000S, Leica, Wetzlar, Germany), maintained in a moist air–liquid (ACSF) interface chamber at room temperature (24–25 °C), and allowed to recover for at least 90 min. LC was identified as a transparent, long, oval area located rostral to the floor of the 4th ventricle and beneath the superior cerebellar peduncle.

### Electrophysiology

Brainstem slices were transferred to a perfusion chamber mounted on an upright microscope (BX51WI, Olympus Optical Co., Ltd., Tokyo, Japan) and continuously perfused with oxygenated ACSF at 2–3 ml min^−1^. Neurons were viewed using a digital camera (C10600 ORCA-R2, Hamamatsu, Japan). Patch pipettes, pulled from borosilicate glass tubing (outer diameter, 1.5 mm; wall thickness, 0.32 mm; Warner Instruments Corp., Hamden, CT, USA), showed a resistance of 3–5 MΩ when filled with the pipetting solutions. All experiments were recorded using a patch amplifier (Multiclamp 700 B; Axon Instruments Inc., Union City, CA, USA). Signals were low-pass filtered at 2 kHz and digitized at 10 kHz using Micro 1401 + Spike2 (Cambridge Electronic Design, Cambridge, UK). For intracellular recordings, the pipette solution constituted (in mM) 131 K-gluconate, 20 KCl, 10 HEPES, 2 EGTA, 8 NaCl, 2 ATP, and 0.3 GTP (pH, 7.2–7.3; osmolarity, 300–305 mOsm). For current-clamp recordings, the bridge was balanced, and recordings were only accepted if the recorded neuron showed a membrane potential (Vm) of at least − 45 mV without applying a holding current and if the action potentials (APs) could overshoot 0 mV. For voltage-clamp recording, Vm was clamped to − 70 mV, unless otherwise specified. For cell-attached voltage-clamp recording, spontaneous AP recordings were made at 29–31 °C. The pipette solution was replaced by normal ACSF [24, 25]. During the electrophysiological recordings, 5 mM kynurenic acid (Sigma, St Louis, MO, USA) and 0.1 mM picrotoxin (Sigma, St Louis, MO, USA) were added in bathing ACSF to block glutamatergic and GABAergic synaptic transmission. For the voltage-clamp recording of A-type K^+^ currents (I_A_), 1 μM tetrodotoxin (Tocris Cookson, Bristol, UK) was mixed in calcium-free bathing ACSF to avoid contamination of the Na and Ca currents.

### Evaluation of developmental reflexes

Developmental reflexes, including surface righting, bar holding, and negative geotaxis, were tested to evaluate muscle tone and physical endurance in mice. Three cohorts of male mice were used in the evaluation of developmental reflexes from postnatal days 3 to 13. The experiments were conducted according to previously reported protocols [26]. To evaluate surface righting, mice pups were gently placed on their backs, and the time taken for them to turn onto their bellies was recorded. To evaluate bar holding, mice pups were lifted by the trunk and held close to a thin metal bar. The period of hanging by their front paws was recorded. To evaluate cliff avoidance, mice pups were placed on the edge of a wooden platform with their noses and forefeet over the edge. The period taken by each pup to move away from the cliff by backing up or by turning sideways was recorded.

### Evaluation of respiration

The tested mice were placed in a whole-body plethysmograph (EMKA Technologies, Paris, France) for 60 min under normocapnic gas (78.5% N_2_, 21% O_2_, and 0.5% CO_2_) with a ventilation pump. Hypercapnic gas (72% N_2_, 21% O_2_, and 7% CO_2_) was applied for 20 min, followed by a period of recovery under normocapnic gas for 20 min. Apnea was manually measured and was defined as the absence of at least two inspirations [27]. The ventilatory parameters, including tidal volume, minute volume, and frequency of breathing, were determined using IOX v2 (EMKA Technologies, Paris, France).

### Data and statistical analyses

Statistical analyses were performed using SPSS version 19.0 for Windows (IBM, Chicago, IL, USA). All data were presented as mean and standard error of mean. To assess the significance of independent variables in WT and Ndn +m/−p mice, Student’s *t* test was used. The level of significance was set at *P* < 0.05.

## Results

### Electrophysiological properties of LC neurons

**Fig. 1:**
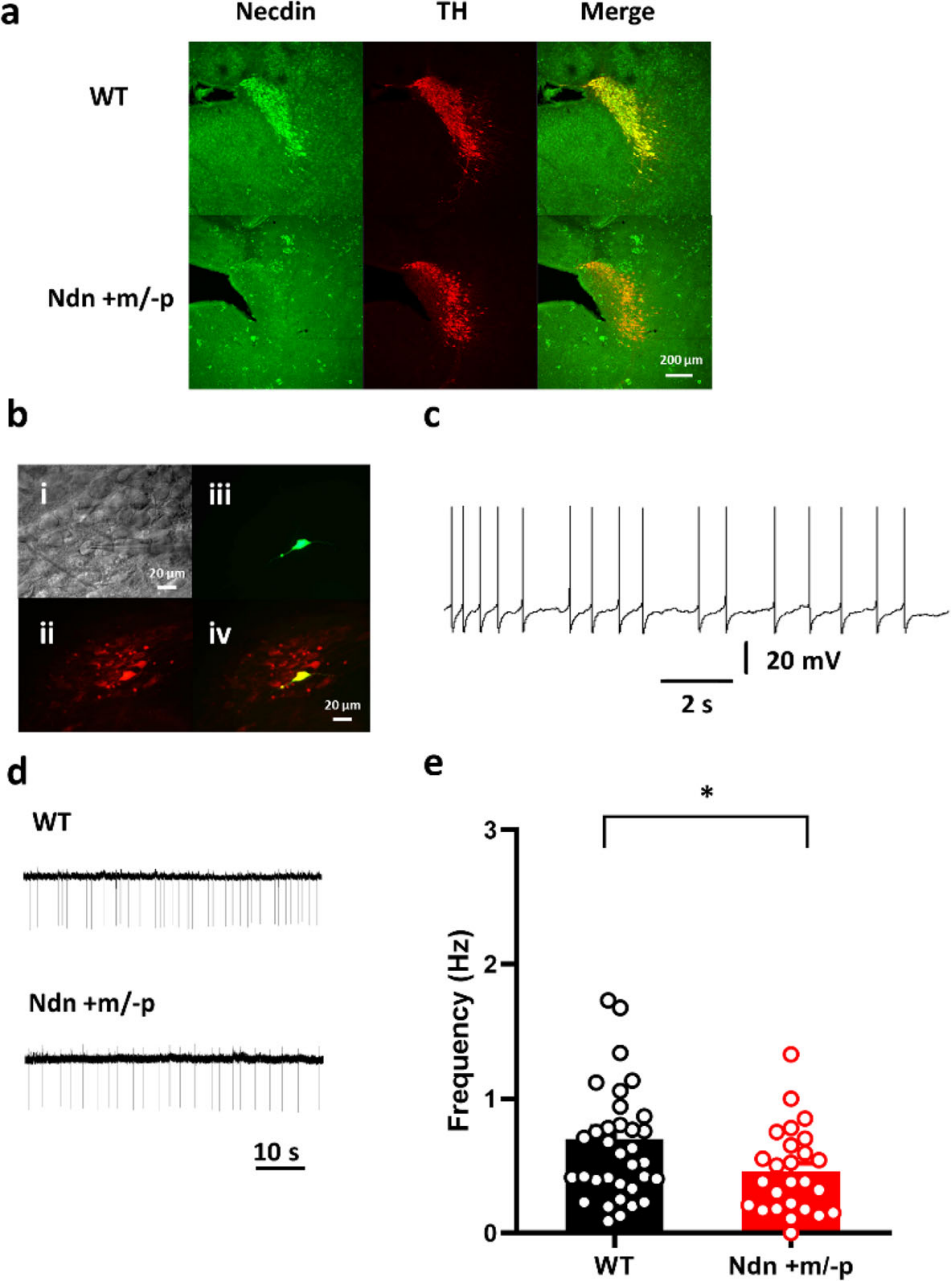
Recording of noradrenergic neurons in the locus coeruleus (LC) of wild-type (WT) and Ndn +m/−p mice. **a** Representative images of LC norepinephrine (NE) neurons of WT and Ndn +m/−p mice. Necdin was abundant in the LC-NE neurons of WT mice. **b** Identification of LC neurons in a sagittal brainstem slice. A non-fixed slice with a recording of LC neurons at high magnification (i). Fluorescence microscopy photographs of a brainstem slice stained with anti-tyrosine hydroxylase (TH) antibody (ii), biocytin (iii), and the merged image (iv). **c** A representative current recording from the LC-NE neuron showed spontaneous firing of action potentials (APs). **d** Representative tracings of spontaneous APs of LC-NE neurons. **e** The spontaneous AP frequency was significantly lower for LC-NE neurons in Ndn +m/−p (*n* = 26 cells from 11 animals) than those in WT mice (*n* = 35 cells in 12 animals). Statistical analyses were performed using Student’s *t* test. Data are presented as mean ± SEM, *P < 0.05

**Fig. 2:**
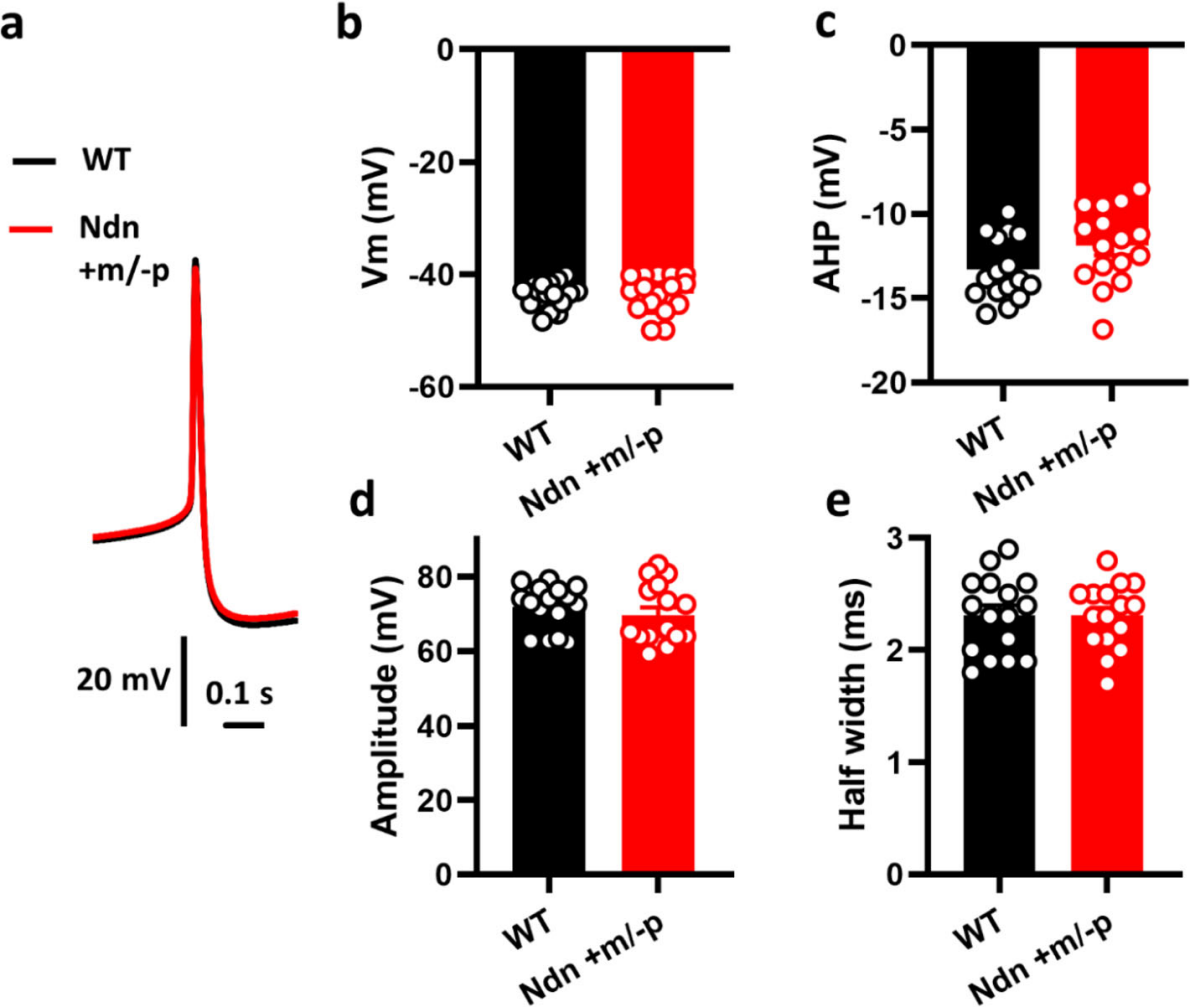
Action potential (AP) morphology of noradrenergic neurons in the locus coeruleus (LC) of wild-type (WT) and Ndn +m/−p mice. **a** Representative spontaneous AP morphology of LC-NE neurons. **b**–**e** The resting membrane potentials (**b**) after-hyperpolarization amplitude (AHP) (**c**), AP amplitude (**d**), and AP half width (**e**) showed no differences for LC-NE neurons of WT (*n* = 16 cells from 12 animals) and Ndn +m/−p (*n* = 16 cells from 11 animals) mice, although there was a trend that the AHP of Ndn +m/−p mice was smaller than that of WT mice. Statistical analyses were performed using Student’s *t* test. Data are presented as mean ± SEM

First, we examined necdin expression in the brainstem of P7 mice. Necdin was abundant in the LC neurons of the WT mice; in Ndn +m/−p mice, there was no residual necdin (Fig. 1a). Next, we evaluated the electrophysiological properties of LC neurons. At high magnification, numerous large neurons could be identified for recording, and the recording cells (biocytin-positive) were also immunoreactive with an anti-TH antibody, documenting these as NE neurons (Fig. 1b). Under current-clamp recording, LC neurons could spontaneously fire APs (Fig. 1c), which is consistent with the results of our previous study [28]. As intracellular recordings unavoidably cause cell damage and possibly affected neuronal spontaneous activities, we evaluated LC neuronal spontaneous activity using only the cell-attach method. Using extracellular recording, we compared the spontaneous firing rates (SFRs) of LC neurons from the WT (*n* = 35 cells from 12 animals) and Ndn +m/−p mice (*n* = 26 cells from 11 animals) and found that Ndn +m/−p mice presented with a significantly slower SFR, which indicated abnormal LC function. The SFRs of WT and Ndn +m/−p mice were 0.70 ± 0.09 and 0.46 ± 0.06 Hz, respectively (*P* = 0.03, Fig. 1e). Under current-clamp recording, we compared the spontaneous AP morphology between WT (*n* = 16 cells from 12 animals) and Ndn +m/−p (*n* = 16 cells from 11 animals) mice (Fig. 2a). The resting membrane potential, post-hyperpolarization amplitude, AP amplitude, AP threshold, and AP half width were not significantly different between the two groups, although there was a trend that post-hyperpolarization amplitude of LC neurons of Ndn +m/−p mice were relatively smaller than those of WT mice (− 11.9 ± 0.6 vs. − 13.3 ± 0.5 mV, *P* = 0.09, Fig. 2b–e).

### Excitability of LC neurons in Ndn +m/−p mice

**Fig. 3:**
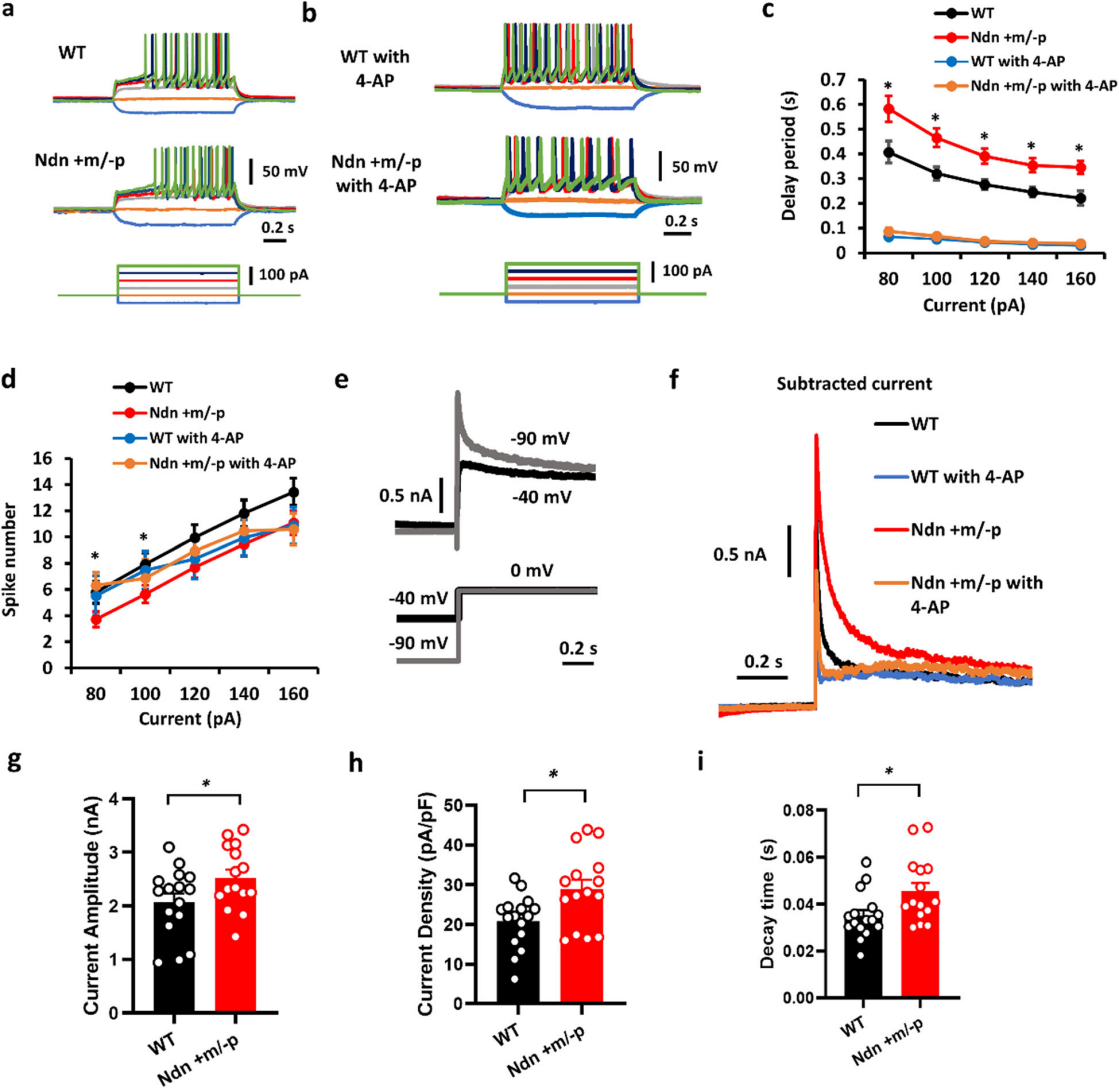
Current- and voltage-clamp recordings in LC-NE neurons. **a** Representative tracings of transmembrane voltage responses from hyperpolarizing to suprathreshold current injections revealed a significant delay in the action potential (AP) firing latency of LC-NE neurons in Ndn +m/−p mice. **b** The delay in the AP firing of LC-NE neurons in both WT and Ndn +m/−p mice can be eliminated using 4-aminopyridine (4-AP). The representative tracings were from the different neurons of **a**. **c** The delay in the AP firing latency from current injections was significantly increased in LC-NE neurons in Ndn +m/−p mice (*n* = 17 cells from 12 animals) than in those in WT mice (*n* = 16 cells from 13 animals). AP firing delay in both WT (*n* = 5 cells from 4 animals) and Ndn +m/−p mice (*n* = 5 cells from 3 animals) decreased after applying 5 μM of 4-aminoantipyrine (4-AP) in the bathing ACSF. Asterisks indicate a significant difference between WT and Ndn +m/−p. Statistical analyses were performed using Student’s *t* tests between WT and Ndn +m/−p mice. **d** AP numbers elicited by current injection were lower in Ndn +m/−p mice subjected to 80-pA and 100-pA current injection, and the difference was eliminated by 4-AP. Asterisks indicate a significant difference between WT and Ndn +m/−p. Statistical analyses were performed using ANOVA with LSD posthoc analysis. **e** Recording of *I*_A_ in LC-NE neurons. Representative tracings illustrated the effects of voltage stepping from − 90 to 0 mV (gray line, *I*_A_ + *I*_DR_) and from − 40 to 0 mV (black line, I_DR_). **f** Subtraction of the two currents yielded the fast activated and inactivated *I*_A_ current; representative tracings of *I*_A_ currents of LC-NE neurons in the Ndn +m/−p (red line) and WT (black line) mice. The *I*_A_ currents of the Ndn +m/−p (orange line) and WT (blue line) mice can be suppressed by 4-AP. **g**–**i** The *I*_A_ current (**g**), current density (**h**), and decay time constant (**i**) were increased in the LC-NE neurons in Ndn +m/−p (*n* = 15 cells from 10 animals) than in those in WT mice (*n* = 16 cells from 9 animals). Statistical analyses were performed using Student’s *t* test. Data are presented as mean ± SEM

As previously reported, on injection of hyperpolarizing current pulses, LC neurons showed delayed firing of APs, and the duration of the delay was voltage dependent [28]. Interestingly, while currents were injected into LC-NE neurons at 20-pA steps to depolarize the neuron, with the membrane potential held at − 70 mV, the delay in AP firing was prolonged significantly in LC-NE neurons of Ndn +m/−p (*n* = 17 cells from 12 animals) than WT mice (*n* = 16 cells from 13 animals, Fig. 3a, c). The APs of LC-NE neurons of Ndn +m/−p mice were also lower than those of WT mice subjected to 80- and 100-pA current injections (Fig. 3d). These findings indicate that LC neurons of the Ndn +m/−p mice showed significantly less excitability than those of WT mice.

Because delays in the firing of APs in LC neurons have been posited to be caused by A-type K^+^ currents (*I*_A_) [28, 29], we applied 5 μM 4-aminoantipyrine (4-AP) in the bathing ACSF during hyperpolarizing current pulses to LC neurons of both WT (*n* = 5 cells from four animals) and Ndn +m/−p mice (*n* = 5 cells from three animals) and found that the delay in the firing of APs decreased (Fig. 3b, c) and that the difference in AP decreased between 80- and 100-pA current injection (Fig. 3d). Therefore, we postulated that prolonged delay in the firing of APs in the LC neurons of Ndn +m/−p mice was due to increased *I*_A_. Using an established protocol, we evaluated I_A_ in LC neurons of WT and Ndn +m/−p mice [30]. Under voltage-clamp recording, outward currents were evoked in LC neurons by stepping the Vm up from − 90 to 0 mV. These outward currents were a mixture of fast activation/inactivation (*I*_A_) and delay rectifier (*I*_DR_) K^+^ currents (Fig. 3d). Voltage stepping from − 40 to 0 mV only evoked the non-inactivated *I*_DR_ currents (Fig. 3d). Subtraction of this *I*_DR_ current from that evoked on stepping from − 90 to 0 mV (*I*_A_ + *I*_DR_) yielded fast activated/inactivated *I*_A_ currents which can be suppressed by 4-AP (Fig. 3e). The properties of LC neurons including series resistance, input resistance, and capacitance in this experiment were demonstrated in supplementary table 1. As predicted, the I_A_ current amplitude and current density were both higher for LC neurons of Ndn +m/−p mice. The *I*_A_ current amplitude of LC neurons of Ndn +m/−p (*n* = 15 cells from 10 animals) and WT (*n* = 16 cells from 9 animals) mice was 2.52 ± 0.15 and 2.07 ± 0.16 nA (*P* = 0.049, Fig. 3f), respectively, and the *I*_A_ current density of Ndn +m/−p and WT mice was 28.80 ± 2.44 and 20.26 ± 1.67 pA/pF, respectively (*P* = 0.012, Fig. 3g). In addition, the decay time constants of *I*_A_ in LC-NE neurons of Ndn +m/−p mice were higher than those of WT mice (Fig. 3h). Collectively, the enhanced and prolonged *I*_A_ decreased the excitability of the LC neurons in Ndn +m/−p mice and possibly led to impaired spontaneous firing of APs in these neurons.

### Neonatal phenotypes of Ndn +m/−p mice

**Fig. 4:**
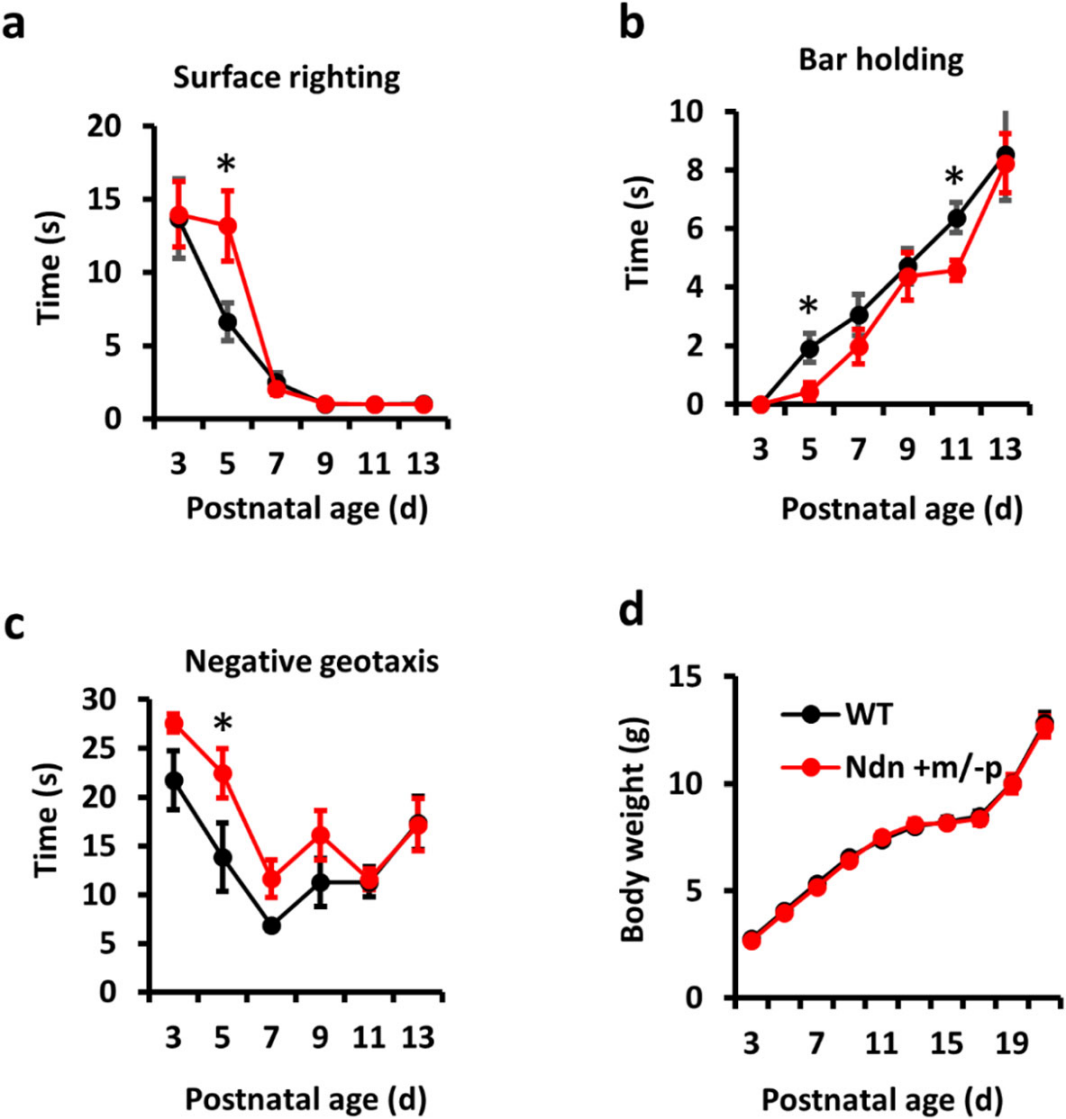
Developmental reflexes and body weight of wild-type (WT) and Ndn +m/−p mice. **a**–**c** Transient hypotonia was observed in Ndn +m/−p mice (*n* = 10 animals) than WT (*n* = 8 animals) by increased latencies of surface righting (**a**) and negative geotaxis reflexes (**c**) and decreased latencies of bar holding (**b**) at postnatal day (P) 5. **d** WT (*n* = 13 animals) and Ndn +m/−p mice (*n* = 14 animals) showed equivalent body weight gain from postnatal days 3 to 21. Statistical analyses were performed using Student’s *t* test. Data are presented as mean ± SEM

As previously reported, *Ndn*^*tm1ky*^ +m/−p mice showed low postnatal lethality, which is similar to neonates with PWS [31]. We evaluated developmental reflexes and respiration in WT and *Ndn* +m/−p mice, which are symptoms that occur in neonates with PWS during the neonatal stage. Muscle tone and physical endurance were assessed by surface righting and bar holding in 8 WT and 10 Ndn +m/−p male mice pups. In Ndn +m/−p mice, surface righting and front limb bar holding were slightly impaired at postnatal day (P) 5 and improved thereafter. At P5, Ndn +m/−p mice showed a two-fold longer latency of surface righting (6.6 ± 1.3 s for WT and 13.2 ± 2.4 s for Ndn +m/−p; *P* = 0.039) as well as shorter front limb bar holding latency (1.9 ± 0.5 s for WT and 0.4 ± 0.3 s for Ndn +m/−p; *P* = 0.016) (Fig. 4a, b), and after P5, Ndn +m/−p and WT mice had similar latencies of surface righting. In addition, Ndn +m/−p mice showed increased latency for negative geotaxis reflex, which is possibly correlated with impaired vestibular proprioceptive function or hypotonia (Fig. 4c) [32]. Because muscle tone and physical endurance are positively correlated with body weight, these were recorded in 13 WT and 14 Ndn +m/−p mice from P3 to P21; the increase in body weight was similar in both groups (Fig. 4d).

**Fig. 5:**
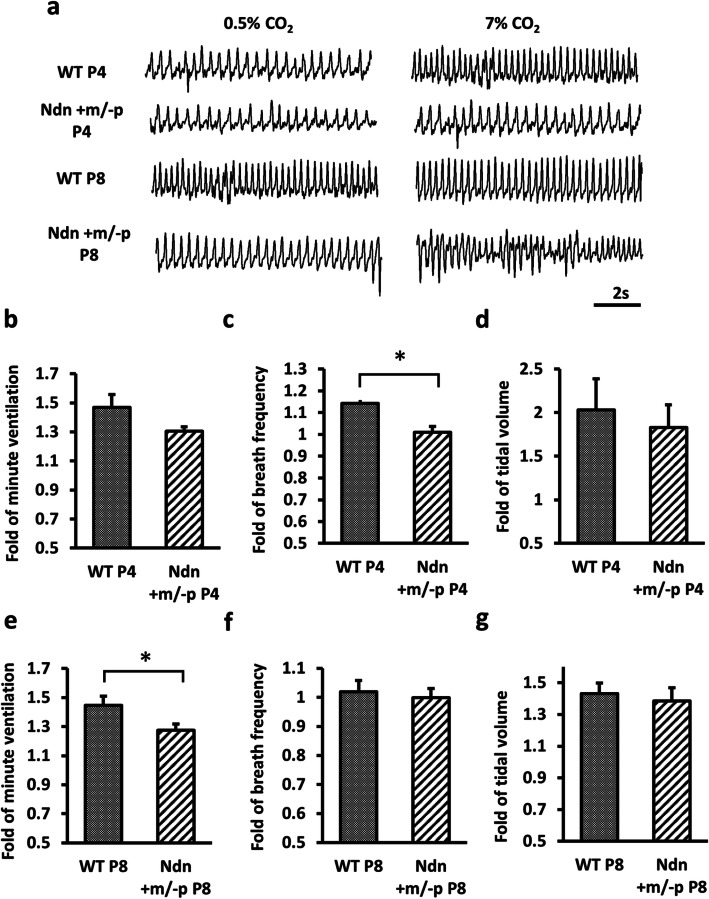
Respiratory responses to hypercapnia in WT and Ndn +m/−p mice. **a** Representative tracing of plethysmography data for WT and Ndn +m/−p mice at postnatal days (P) 4 and 8. **b**–**d** Ventilatory responses to hypercapnia in Ndn +m/−p and WT mice (*n* = 8 for both groups) at P4 revealed that WT mice had significantly increased breathing frequency while breathing 7% CO_2_ (**c**). **e**–**g** Ventilatory responses to hypercapnia in Ndn +m/−p and WT mice (*n* = 11 for both groups) at P8 revealed that WT mice had significantly increased minute ventilation while breathing 7% CO_2_ (**e**). Statistical analyses were performed using Student’s *t* test. Data are presented as mean ± SEM

Because patients with PWS presented with blunted respiratory responses to hypercapnia in previous studies [21, 33] and because LC neurons enhanced CO_2_ sensitivity [18], we examined whether necdin deficiency recapitulated respiratory phenotypes using in vivo plethysmography at P4 (*n* = 8 for both WT and Ndn +m/−p mice) and P8 (*n* = 11 for both WT and Ndn +m/−p mice). We found no differences in baseline respiratory parameters, including tidal volume, breathing frequency, minute ventilation, and apnea counts, between the two groups. We evaluated respiratory responses to hypercapnia at P4 and P8. Hypercapnia increased minute ventilation in both WT and Ndn +m/−p mice, although this increase was impaired in Ndn +m/−p mice at both P4 and P8 (Fig. 5b, e). Minute ventilation increases during hypercapnia were 1.31 ± 0.03-fold and 1.26 ± 0.00-fold in Ndn +m/−p mice at P4 and P8, respectively, and 1.47 ± 0.09-fold (*P* = 0.115 compared with Ndn +m/−p mice, Fig. 5b) and 1.45 ± 0.06-fold (*P* = 0.024 compared with Ndn +m/−p mice, Fig. 5e) in WT mice at P4 and P8, respectively. Interestingly, the hypercapnic conditions increased breathing frequency in WT mice at P4 but not at P8 (Fig. 5c, f). For Ndn +m/−p mice, breathing frequency remained unchanged during hypercapnia at both P4 and P8 (Fig. 5c, f). At P4, the changes in breathing frequency in WT and Ndn +m/−p mice during hypercapnia were 1.14 ± 0.06-fold and 1.01 ± 0.03-fold, respectively (*P* = 0.035, Fig. 5c), suggesting that Ndn +m/−p mice showed defective CO_2_ chemosensitivity during the neonatal period. Hypercapnia increased the tidal volume in both WT and Ndn +m/−p mice at both P4 and P8 (Fig. 5d, g); although the increase was impaired in Ndn +m/−p mice, the differences were not significant.

## Discussion

Our study revealed that LC noradrenergic neurons of *Ndn*^*tm1ky*^ mice showed significantly decreased spontaneous activities as well as impaired excitability, which were mediated by enhanced A-type voltage-dependent potassium currents. *Ndn*^*tm1ky*^ mice also possessed some neonatal phenotypes possibly related with LC-NE dysfunction, such as transient hypotonia and poor ventilatory responses to hypercapnia. Our data suggest that LC—the primary source of norepinephrine in CNS—is involved in PWS pathogenesis.

Previously, the hypothalamus had been the region of interest for determining PWS pathophysiology because of the endocrinological manifestations of the disease, which include hyperphagia, obesity, hypogonadism, and growth hormone deficiency [34, 35]. However, hypothalamus and pituitary gland dysfunction do not correlate with central apnea and hypotonia, which are important risk factors for premature death in infants and children with PWS [14, 36]. Therefore, this study focused on another important region in the brainstem—LC—using necdin-deficient mice. Most previous studies have focused on respiratory abnormalities in necdin-deficient mice because of their high postnatal lethality following respiratory instability [10, 12, 37, 38]. Other behavior-related data of these mice, however, are lacking. Our study demonstrated that, in addition to breathing, Ndn^tm1ky^ mice show defective postnatal motor development, rendering this mouse strain a suitable platform for investigating the neurobiological mechanisms underlying the neonatal symptoms of PWS.

Necdin, transcribed from the paternal Ndn allele in both mice and humans, is a neural differentiation-associated protein and is expressed exclusively in postmitotic neurons of the CNS and peripheral nervous system [39, 40]. Our study demonstrated LC dysfunction in Ndn^tm1ky^ mice and corroborated the previous histological findings of Pagliardini et al. who used another necdin-deficient mouse model (Ndn^tm2stw^) to reveal the abnormal distribution and appearance of noradrenergic neurons in the medulla [10]. Considering the neonatal phenotypes of necdin-deficient mice, including hypotonia and blunted respiratory responses, the noradrenergic system dysfunction in both Ndn^tm1ky^ and Ndn^tm2stw^ mice may be an important etiology of neonatal PWS. Therefore, in this study, we evaluated the electrophysiological properties of LC neurons in Ndn^tm1ky^ mice. First, the SFR of LC neurons was lower in the necdin-deficient mice. LC is important for attention and behavior due to its functional switch between the phasic and tonic modes of output [41]. Thus, the decreased SFR of LC neurons might affect the vital function of the LC-NE system in Ndn^tm1ky^ mice. Next, we analyzed the spontaneous AP morphology of LC neurons in Ndn^tm1ky^ mice but found no significant differences compared with WT mice. AP morphology is determined primarily by the dynamics of voltage-gated sodium and potassium channels, which are also associated with the provocation of seizures [42]. This electrophysiological finding is consistent with the clinical observation that patients with PWS scarcely develop seizures [43]. Finally, the latency of the first AP firing following the injection of current into LC-NE neurons of Ndn^tm1ky^ mice was significantly delayed. This delayed AP firing latency was due mostly to A-type potassium currents, which participate in neuronal dendritic calcium signaling, signal integration, and synaptic plasticity [44]. In the brainstem noradrenergic neurons of the A7 catecholamine cell groups, A-type potassium currents play important roles in the regulation of the shape and firing frequency of APs as well as in synaptic integration [30]. Our data indicate that necdin deficiency increases A-type potassium currents in LC-NE neurons, decreases their excitability, and possibly decreases SFR. Therefore, the baseline phasic activity of LC-NE neurons in Ndn^tm1ky^ mice may be affected.

Our study revealed no respiratory insufficiency and postnatal lethality in the necdin-deficient *Ndn*^*tm1ky*^ mice, consistent with a previous literature [13]; however, the *Ndn*^*tm1ky*^ mice still exhibited blunt ventilatory responses to hypercapnia, indicating respiratory pathology. Other necdin-deficient mice, such as those with strains *Ndn*^*tm1stw*^ and *Ndn*^*tm2stw*^, manifested severe congenital respiratory insufficiency and high postnatal lethality, which are associated with unstable respiratory drive caused by pre-Bötzinger complex (pre-BötC) dysfunction [37, 45]. The primary source of rhythmic inspiratory–excitatory drive is the pre-BötC located in the ventral respiratory column of the brainstem [46]. Although necdin mRNA is highly expressed in the ventrolateral medulla, including the pre-BötC [37], the morphology of NK1R-positive cells indicating pre-BötC neurons appeared normal in *Ndn*^*tm2stw*^ mice [10]. Conversely, the morphology and architecture of neurons in the surrounding medullary structures that provided a conditioning synaptic input to the pre-BötC neurons were abnormal; these neurons included enlarged and abnormally oriented serotonergic neurons and aberrant noradrenergic neurons [10]. During ontogeny, central chemoreception is necessary for respiratory rhythm stimulation [47]; hence, the abnormal serotonergic and noradrenergic neurons possibly corresponded to the abnormalities of the pre-BötC neuronal activity and respiratory drive in necdin-deficient mice. Reportedly, the morphology and activity of brainstem serotonergic neurons are aberrant in necdin-deficient mice, possibly induced by an increase in serotonin transporter [11, 12]. In our study, the necdin-deficient mice exhibited brainstem noradrenergic dysfunction. We also added that LC-NE dysfunction is possibly involved in the respiratory pathology of such mice.

We provide evidence that necdin deficiency—a universal presentation of PWS—leads to dysfunction of the LC-NE system. In addition to its roles in arousal and cognitive processes, the LC-NE system is the primary responder to stress in CNS [41, 48, 49]. Chronic stress leads to long-lasting hyperactivity and increased sensitivity of LC neurons as well as the excessive activity of NE neurons under stress, which lead to stress-induced deficits in cognitive functions depending on the prefrontal cortex [49]. LC hyper-responsiveness has been observed in patients with post-traumatic stress disorder [50], which manifests as significant arousal and emotional symptoms of angry outbursts. Such symptoms are also frequently found in patients with PWS [51]. Moreover, psychiatric symptoms including temper tantrums and aggressive behaviors significantly interfere with the quality of life of patients with PWS [52].

There are some limitations to this study. First, we could not attain the causality between neonatal phenotypes and impaired LC-NE function in this observational study. Second, *Ndn*^*tm1ky*^ +m/−p mice were deficient in necdin in the whole body, and we cannot exclude the possibility that hypotonia and blunted ventilatory responses to hypercapnia were induced by myopathy or peripheral chemosensory failure. However, this is the first study to demonstrate LC-NE neuronal dysfunction in necdin-deficient mice, a widely used PWS animal model, and indicate that LC-NE dysfunction is possibly involved in the pathogenesis of some PWS symptoms. Using optogenetic or chemogenetic experiments, the cell type-specific neuronal firing activity can be modulated, and the causality between neuronal activity and physiological function can be evaluated, but these approaches are difficult to use for neonatal phenotypes in the present study. However, to our knowledge, LC neurons play important roles for ventilatory responses to hypercapnia and muscle toning [20, 53]. Other psychiatric or behavioral phenotypes of PWS that develop in adolescence or adulthood, such as temper tantrums and aggressive behaviors, will be good targets to evaluate the causal relationship between the LC-NE dysfunction and PWS phenotypes.

## Conclusions

In conclusion, this study demonstrated that Ndn^tm1ky^ mice exhibit neonatal symptoms of PWS: hypotonia and impaired ventilatory responses to hypercapnia. Moreover, our electrophysiological experiments indicated abnormal spontaneous and evoked activities of LC neurons in Ndn^tm1ky^ mice, mediated by enhanced *I*_A_ currents. With a better understanding PWS pathogenesis, a mechanistic approach can be taken for developing treatments of PWS-related clinical symptoms.

## Supplementary information

**Additional file 1:.** Table S1. The series resistance, input resistance, and capacitance of voltage-clamp experiments measuring IA currents

## Data Availability

The datasets supporting the conclusions of this article are available in the Synapse repository (https://www.synapse.org/#!Synapse:syn21625611/files/).

## Abbreviations

ACSF: Artificial cerebrospinal fluid
AP: Action potential
CNS: Central nervous system
PWS: Prader–Willi syndrome
LC: Locus coeruleus
PB: Phosphate-buffer
SFR: Spontaneous firing rates
TH: Tyrosine hydroxylase
WT: Wild-type
