# Supplementary material for: Firing activity of locus coeruleus noradrenergic neurons decreases in necdin-deficient mice, an animal model of Prader–Willi syndrome
Source: J Neurodev Disord. 2020 Jul 29;12:21. doi: 10.1186/s11689-020-09323-4 (PMC7389383; doi:10.1186/s11689-020-09323-4)
Supplement: Supplementary file 1 — Additional file 1:. Table S1. The series resistance, input resistance, and capacitance of voltage-clamp experiments measuring IA currents [file 11689_2020_9323_MOESM1_ESM.docx]

**Supplementary table 1.** The series resistance, input resistance, and capacitance of voltage-clamp experiments measuring IA currents

|  | WT | Ndn +m/-p | p value |
| --- | --- | --- | --- |
| Series Rs (Mohm) | 24.01±2.87 | 20.218±1.39 | 0.25 |
| Input Rs (Mohm) | 658.31±61.9 | 722.7±294.76 | 0.57 |
| Series/input Rs | 0.046±0.009 | 0.034±0.004 | 0.27 |
| Capacitance (pF) | 102.99±5.99 | 94.04±7.06 | 0.34 |

Values are expressed as mean ± SEM

Abbreviations: WT, wildtype; Rs, resistance
